# An FEP Microfluidic Reactor for Photochemical Reactions

**DOI:** 10.3390/mi9040156

**Published:** 2018-03-30

**Authors:** Tomasz Szymborski, Paweł Jankowski, Dominika Ogończyk, Piotr Garstecki

**Affiliations:** 1 Institute of Physical Chemistry, Polish Academy of Sciences, Kasprzaka 44/52, 01-224 Warsaw, Poland; tszymborski@ichf.edu.pl (T.S.); dogonczyk@ichf.edu.pl (D.O.); 2 Soft Materials Laboratory, Institute of Materials, Ecole Polytechnique Fédérale de Lausanne, 1015 Lausanne, Switzerland

**Keywords:** fluorinated ethylene propylene (FEP), microreactor, photochemistry, organic synthesis

## Abstract

Organic syntheses based on photochemical reactions play an important role in the medical, pharmaceutical, and polymeric chemistry. For years, photochemistry was performed using high-pressure mercury lamps and immersion-wells. However, due to excellent yield, control of temperature, selectivity, low consumption of reagents and safety, the microreactors made of fluorinated ethylene propylene (FEP) tubings have recently been used more frequently. Fluoropolymers are the material of choice for many types of syntheses due to their chemical compatibility and low surface energy. The use of tubing restricts the freedom in designing 2D and 3D geometries of the sections of the microreactors, mixing sections, etc., that are easily achievable in the format of a planar chip. A chip microreactor made of FEP is impracticable to develop due to its high chemical inertness and high melting temperature, both of which make it difficult (or impossible) to bond two plates of polymer. Here, we demonstrate a ‘click’ system, where the two plates of FEP are joined together mechanically using a tenon and a mortise. The concept was presented by us previously for a preparation polytetrafluoroethylene (PTFE) microreactor (Szymborski et al. *Sensors Actuators, B Chem.* 2017, doi:10.1016/j.snb.2017.09.035). Here, we use the same strategy for FEP plates, test the use of the chips in photochemistry and also describe a custom-designed non-transparent polyethylene (PE) mask-holder with a circular opening to guide and focus the ultraviolet (UV) illumination. The solutions that we describe offer tight microreactor chips, preventing any leakage either of the liquid reagents or of UV light outside the reactor. This allows for conducting photochemical synthesis without a fume hood and without special protection against UV radiation.

## 1. Introduction

In the past years, there has been a significant increase in the use of continuous flow reactors in organic syntheses [1,2,3,4,5]. A variety of reactors have been demonstrated in the synthesis of numerous organic reactions [6]. One of the fastest areas of development is photochemical organic synthesis [1,7]. These reactions have found a wide application in the fields of pharmaceutical, polymer, analytical and medicinal chemistry. For many years, bath reactors were used frequently both in industry and academia. Two general types of bath reactors are commonly used: immersion-well and chamber reactors. The first one uses a single low, medium or high-pressure mercury (Hg) lamp placed in the very center of double-walled immersion well: the reaction medium surrounds the Hg lamp in a separate vessel, which makes the process effective. Chamber reactors use an external array of fluorescent tubes with internal reaction flasks (e.g., flash tubes or Schlenk flasks). This configuration of source of light and reaction flasks allows for multiple reaction synthesis at the same time. Both types are widely known and used, despite their disadvantages, e.g., they are big and expensive, parts of a reactor are exposed to strong UV radiation and thermal stresses, thus have to be exchanged regularly, and the immense volume of reagents causes poor mass and heat transfer. Due to these limitations of the bath reactors, coil based reactors (CBR) for photochemistry have become popular [8]. They are usually made of commercially available tubings made of inert fluoropolymers like polytetrafluoroethylene (PTFE), ethylene-tetrafluoroethylene (ETFE) or perfluoroalkoxy copolymer (PFA), and the most popular—fluorinated ethylene propylene (FEP). The tubings typically have an outer diameter of 1/8″ or 1/16″ and various inner diameters (e.g., 0.01″, 0.02″, 0.03″, etc.). The design of CBR usually involves a long FEP tubing wrapped tightly around a cylindrical ultraviolet (UV) lamp. After being mixed, the reagents run through the tube, which has a sufficiently low inner diameter for the light to penetrate through it and interact with reagents. To increase the efficiency of the reactor, multiple layers of the tube can be used [9]. In the CBR photoreactor the residence time of reactants in the tube is determined by the volume of the tubing and the flow of the liquid (parameter set on the syringe pumps). The temperature can be easily controlled by putting tubes inside a thermostatic unit. This type of a microreactor is widely and successfully used in many applications, mostly because it is easy to build with FEP tubes and cost-effective. 

For organic photosynthesis involving UV irradiation, FEP is an ideal material due to its good transmission properties, excellent physical properties (e.g., it can withstand relatively high temperatures and mechanical stresses) and superb chemical resistance to organic solvents. FEP is commercially available in a wide range of diameters, thus its use in photo-reactors is cost-efficient. However, the use of fluorpolymer tube for microreactors has disadvantages. Tube microreactors are prone to clogging, especially if the product precipitates. These reactors are not easy to disassemble for cleaning and general maintenance, e.g., FEP and other polymers degrade after long irradiation times. Finally, high purity polymers have to be used in the leaching of plasticizers from polymer which can have a negative impact of photocatalytic process.

Together with the rise of microfluidics, a new type of flow reactors, known as microfluidic reactors, were developed [10,11]. They consist of two plates of polymer (i.e., polycarbonate (PC) or polymethylmethacrylate (PMMA)), elastomer (i.e., PDMS) or glass with a microchannels cross-section of below 1 mm^2^. The channels are usually micromachinned via computer numerical controlled (CNC) milling machines (PC, PTFE, PMMA and other polymers with high Young module), made via soft photolithography (polydimethylsiloxane (PDMS)) or hot embossing (PMMA, cyclic olefin copolymer (COC)). Usually, bonding of two separate plates of polymer is done at elevated temperatures (i.e., heating both plates to temperature around of melting point of polymer and using high pressure to merge them [12]) or chemical methods, where both plates are modified chemically on the surface to ensure permanent covalent connection [13]. Also, different types of glue can be used to bond two plates [14]. All these methods fail when our material of choice for the microreactor is a fluorinated polymer, i.e., PTFE or FEP. Fluorinated polymers possess high melting temperature (for FEP it is ca. 260 °C). Therefore, thermal bonding can be challenging. They are also chemically inert and possess low surface energy; thus, no chemical modification or gluing is possible. Existing microfabrication techniques suitable for fluoropolymers include synchrotron radiation direct photoetching [15,16], argon ion beam [17,18], or magnetically controlled reactive ion etching [19]. None of these techniques was applied for developing a microfluidic device. Sahlin et al. demonstrated a method of making microfluidic channels with the sizes down to 13 µm in FEP [20]. The technique is based on dual-layer tubing consisting of an outer layer of PTFE and inner layer of FEP. When heated above 350 °C the outer PTFE layer shrinks, whereas the inner layer of FEP melts, thus filling all empty space inside the tubing with FEP. The structure of a channel is formed using tungsten wire as a template, which is pulled out after completion of the shrinking and melting process. This process, however, was used only for the tubing and is not applicable for raw FEP plates.

In our recent publication [21], we have demonstrated a new approach for bonding of materials with high melting temperature or high chemical inertness. We proposed a ‘click’ system, where one plate (male) possess a tenon, whereas a second plate (female) possess a grove. The cross-section of the tenon is rectangular, its height is smaller than the depth of the groove. In addition, the width of the tenon is bigger than the width of the mortise. After placing these two plates together and compressing with a press, the tenon is pushed into the mortise. The channels for the flow of reagents are micromachinned in the male plate, so after mechanical bonding with a press there is a microfluidic channel between plates. To ensure the tightness of the chip, the difference between the width of the tenon and the width of groove should be between 30 µm and 50 µm. Detailed parameters and information about the ‘click’ system can be found in Szymborski et al. [21].

In this paper, we demonstrate the use of a ‘click’ system for development of an FEP based photoreactor. We have used transparent FEP plates with micromachinned ‘click’ system and microchannel for performing organic photosynthesis. Also, we developed a special holder made of non-transparent materials with an opening for UV-lamp or custom-made UV-module. The photoreactor is compressed with a holder and M5 screws, which ensures no leakage and makes no UV radiation available outside the microreactor. Our microreactor is compact (consists of equivalent of 75 cm of FEP tube, yet the whole chip is 32 cm^2^), and easy to disassemble and clean. The use of CNC micromachining allows us to design any microfluidic geometry, which makes it a very versatile system.

Below we provide proof-of-concept demonstrations of two photoreactions: (i) bromination of indanone with N-bromosuccinimide and (ii) thiol-ene reaction. The simple design and properties of FEP-based microreactor (low cost, small dimensions, easy to disassemble and clean) makes them very attractive for photochemical applications.

## 2. Materials and Methods

### 2.1. Materials 

Symalit^®^ FEP was bought from Quadrant Group (Quadrant Group Limited, Burgess Hill, UK) in a form of 5 mm thick plates. For the holder, we used two different materials: the transparent holder (to visualize the flow inside the photoreactor) was cut from a 10 mm thick polycarbonate (PC) sheet Makrolon^®^ (Covestro Plastics, Leverkusen, Germany). The non-transparent holder (for making photochemical synthesis) was made from an extruded 10 mm PE sheet, from Plastics Group Sp. z o.o. (Warsaw, Poland).

Substrates for organic synthesis: 1-indanon, N-bromosuccinimide, 2,4,6-tris(allyloxy)-1,3,5-triazine, methyl mercaptoacetate and diphenyl(2,4,6-trimethylbenzoyl)phosphine oxide were bought from Sigma-Aldrich (St. Louis, MO, USA) and used as received, without additional purification. Acetonitryle (HPLC grade) was bought from POCh S.A. (Gliwice, Poland).

Products were analyzed with high performance liquid chromatography (HPLC); refractive index detector (RID) RID-6A and ultraviolet–visible spectroscopy (UV/Vis) SPD-6A, both Shimadzu (Shimadzu Corporation, Kyoto, Japan) and column Kromasil 100-C18 Sigma-Aldrich, 5 µm. The column was 250 × 4 mm with additional precolumn.

### 2.2. Spectral Analysis of Fluorinated Ethylene Propylene (FEP)

First, we checked transparency of FEP plates for UV-Vis radiation at the typical range of wavelength that are used in photochemical reactions. We demonstrate a comparison of transmittance in the function of wavelength for three polymers: polytetrafluoroethylene (PTFE), polycarbonate (PC) and fluorinated ethylene propylene (FEP). For the test we used plates with 2 mm thickness and dimensions 10 mm × 40 mm. After cleaning the surface with isopropanol, the plate was placed in a UV/Vis spectrophotometer (Thermo Scientific™, type Evolution 201, Waltham, MA, USA) and transmittance was measured from 200 nm up to 650 nm. We also measured the density of power of the UV diode we used in the experiments. The measurements were performed with a laser power meter (Ophir, Vega, Ophir Optronics, Jerusalem, Israel) for air, 2 mm thick FEP and 5 mm thick FEP plate (see Supplemental Figures S1–S3 for details).

### 2.3. Preparation of the FEP Microreactor

Fabrication of the microreactor comprised of several steps. We start with a computer aided design (AutoCAD, version 2017, Autodesk, Inc., San Rafael, CA, USA). A design of the microreactor including the ‘click’ system and the channels/holes inside the chip must be made in a CAD or computer aided manufacturing (CAM) software. We used MasterCAM X2 (CNC Software, Inc., Tolland, CT, USA), to design the chip and to prepare the G-code file for the CNC machine (MSG4025, Ergwind, Poland, operated with cncGraf). Figure S4 shows the chip with channels, holes and a ‘click’ system on the edge of the chip. We then cut the FEP plate into a size suitable for a CNC machine holder (120 mm × 140 mm) and cleaned the plate with acetone to remove dust and organic pollutants. We milled the ‘click’ system and the microchannels in a single session without removing the FEP plate from the work table. Afterwards, we drilled holes of 0.80 mm in diameter for the inlets and outlets. After machining we removed the plate, cut the microreactor out from the plate, removed residues of FEP particles with compressed air and washed it with water and acetone. After drying we placed both parts of the microreactor against each other and pressed them together with a hand press. In order to interface the reactor with PTFE/FEP tubing, we placed short steel needles into the ports. 

Due to the low friction coefficient of FEP we used an additional holder with openings for M6 bolts to additionally compress both plates. The holder was CNC machined with black, non-transparent polyethylene (PE) plate (10 mm thick), thus it cuts off the UV light from inside of the microreactor. In the center of the holder we created a circular opening (23 mm in diameter) for placing a commercial UV lamp or custom-made UV light-emitting diode (LED) illumination. 

### 2.4. Leak Tests of the Microreactor

Fluidic tightness of microreactors used for organic synthesis is essential. Firstly, many reactions, especially organometallic, are very sensitive to oxygen and moisture contained in the air. Therefore, it is essential to isolate reactants inside the microreactor from the external environment (i.e., air). Secondly, leaks of the reactants or their products may be hazardous, e.g., BuLi can ignite in contact with the air. Also, products of reaction can be toxic; therefore, nothing should leak from the reactor. We have already thoroughly checked the microreactor with a ‘click’ system for tightness [21]. We verified the tightness of the tenon-grove junction and possible flow between microchannels. The microreactor was placed in the holder which consisted of two plates made of PC (both 10 mm thick). The results for PTFE showed that this junction is very tight, and no flow outside the channels is possible. In this paper, since the FEP is a transparent polymer, to visualize the flow inside the microreactor we used ethanol (99.8%) with Oil Blue and isopropanol with Red Oil to increase the contrast of the liquid inside the microchannel and visualize the flow. That gave us information about the flow inside microchannels and possible leakages to the grove. Additionally, we tested our microreactor for maximum working pressure. To do that we placed the microreactor with tubings into a water container. Then the outlet was closed and we pumped pressurized air to the inlet of the chip. In addition we monitored the pressure using the manometer attached to the inlet of the chip. The air pressure was increased till we observed the unsealing of the microreactor, i.e., the air bubbles. Please see Supplemental Materials, Figure S5 for the picture of the experimental setup.

### 2.5. Photoreactions Using the FEP Microreactor

**Figure 1:**
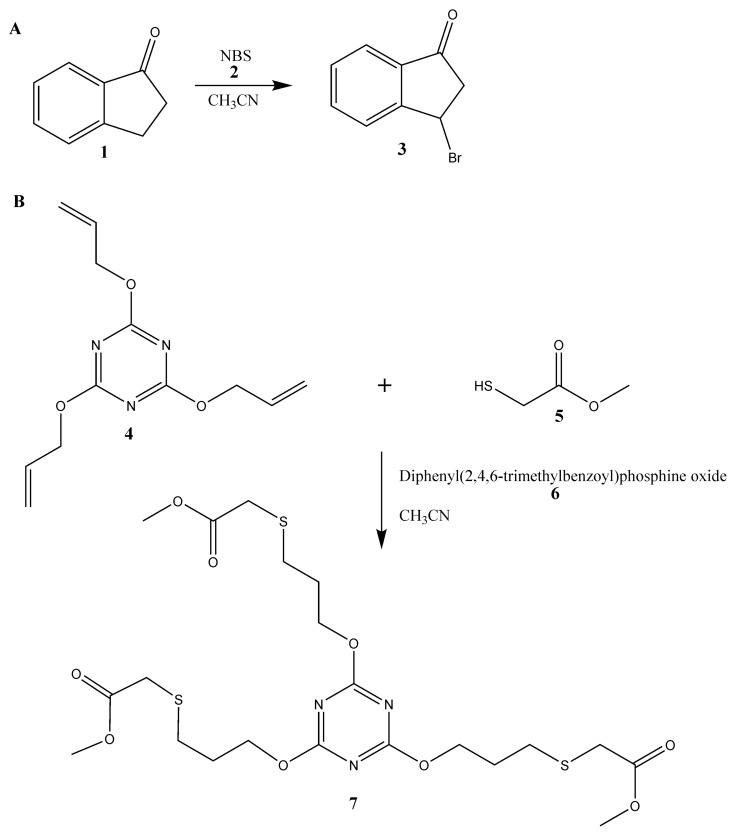
Schemes of carried out in the fluorinated ethylene propylene (FEP) microreactor reactions: bromination (**A**) and thiol-en reaction (**B**).

We give a proof-of-concept by making two photochemical synthesises. The first reaction was bromination of 1-indanon (**1**) with N-bromosuccinimide (**2**) in acetonitrile and second was thiol-ene reaction with the use of 2,4,6-tris(allyloxy)-1,3,5-triazine (**4**), methyl mercaptoacetate (**5**) and diphenyl(2,4,6-trimethylbenzoyl)phosphine oxide (**6**) as an organic photoinitiator. The scheme of synthesis is demonstrated in Figure 1.

## 3. Results and Discussion

### 3.1. FEP Microreactor

**Figure 2:**
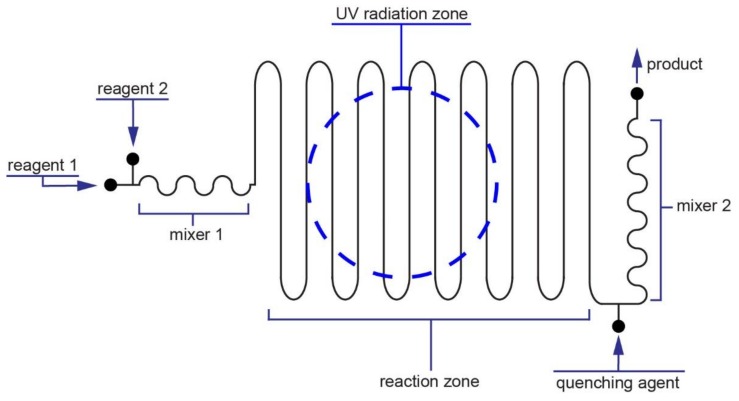
Schematic diagram (without maintaining the scale) of the FEP microreactor used in experiments. Microreactor consists of: two inlets for reagents, mixer for initial mixing reagents, main reactor where ultraviolet (UV) radiation affects reagents, and finally inlet for quenching reagent together with mixer. The precise computer aided manufacturing (CAM) schematic of the microreactor is shown in Figure S4.

We conducted the tests and experiments using an FEP microreactor made of two separate plates, with dimensions 44 mm × 73 mm and 5 mm thick. We used 4.00 mm wide tenon and 3.95 mm groove to mechanically bond two plates, thus making a photochemical reactor. The lumen of the microfluidic channel was 0.6 mm × 1.0 mm. Our photoreactor comprised four main parts (see Figure 2):

(i) Two inlets that combine two streams of reagent 1 and reagent 2;
(ii) The first mixer (mixer 1) which is 82 mm long;
(iii) The main part of the microreactor (aka reaction zone) where the reaction takes place-over; this part of the microreactor is placed UV lamp (UV radiation zone);
(iv) The inlet for a quenching agent, with a 25 mm long section of mixer 2.

The total length of the channel is 752 mm. The first part of the channel is mixer 1 whose task is to preliminary mix the substrates (see Figure S6 for detailed description of the parameters of the mixing). The length of the channel which is exposed to UV radiation is about 50% of the total length. The photoreactor is placed between two plates made of non-transparent 10 mm thick PE. The plates were compressed via eight M6 screws. The compression is essential as the FEP and PTFE possess low friction coefficients; thus, external force is necessary to place the tenon in the mortise during the operation of the microreactor. We micromachined holes in the top PE plate to place the tubings and created one circular opening with diameter of 23 mm for the UV diode lamp. The position of the UV lamp over the microchannels is depicted in Figure 2. The detailed CAD scheme of the microreactor is shown in Figure S4, and the assembled microreactor is shown in Figure S7.

### 3.2. Spectral Analysis of FEP

**Figure 3:**
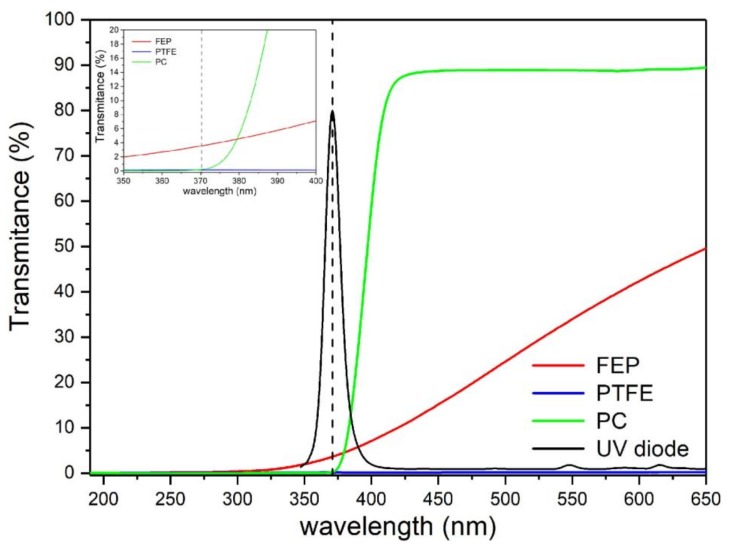
Comparison of transmittance as a function of wavelength for three different 2 mm thick plates of polymers: polytetrafluoroethylene (PTFE) (blue), polycarbonate (PC) (green) and FEP (red). Additionally, we present a UV diode spectrum range (black) that was employed in the model reactions (see Section 3.4). The dash line is main wavelength of the UV diode, λ = 371 nm. The inset demonstrates transmittance of the polymer plates in range 350 nm to 400 nm. The transmittance of the FEP plate is below 4%, whereas the transmittance of PTFE and PC are close to 0%.

The results of spectral analysis for all three polymer plates (FEP, PTFE and PC) are shown in Figure 3. The thickness of all three samples was 2 mm. Additionally, we placed on the plot a UV LED diode spectrum with a main length at 371 nm. This UV diode was used in our further experiments; thus, it was crucial to estimate the transmittance of polymers at this wavelength, especially when FEP is used for the construction of the microreactor. 

The results demonstrate that the polycarbonate (green line) cannot be used as a material for a photoreactor as its transmittance for 371 nm is close to 0% (see inset in Figure 3). A similar result (0% transmittance) is observed for Teflon (PTFE). We observed a decrease of transmittance value below 4% for FEP, a level sufficient for the initiation of photochemical reactions. 

We also measured the density of power (W·cm^−2^) as a function of the power of our UV lamp. The measurement was performed with a dedicated laser power meter (Ophir, Vega, Jerusalem, Israel) where the distance between UV diode and sensor was set to 5 mm. The measurement setup is shown in Figure S1. The density of power as a function of UV lamp (as a percentage of maximal power) is shown in Figure S2. Moreover, Figure S3 reveals the measurement of density of power in a situation where the gap between UV diode and sensor is filled with air, 2 mm thick FEP or 5 mm thick FEP. We showed that the use of the FEP plates (2 and 5 mm) enables constant and fixed permeability of energy (10% and 5%, respectively) for the whole range of power of the UV lamp (inset in Figure S3). Thanks to this, even thicker FEP plate (5 mm) can potentially find applications in photochemical reactions.

### 3.3. Leak Test of FEP Microreactor

**Figure 4:**
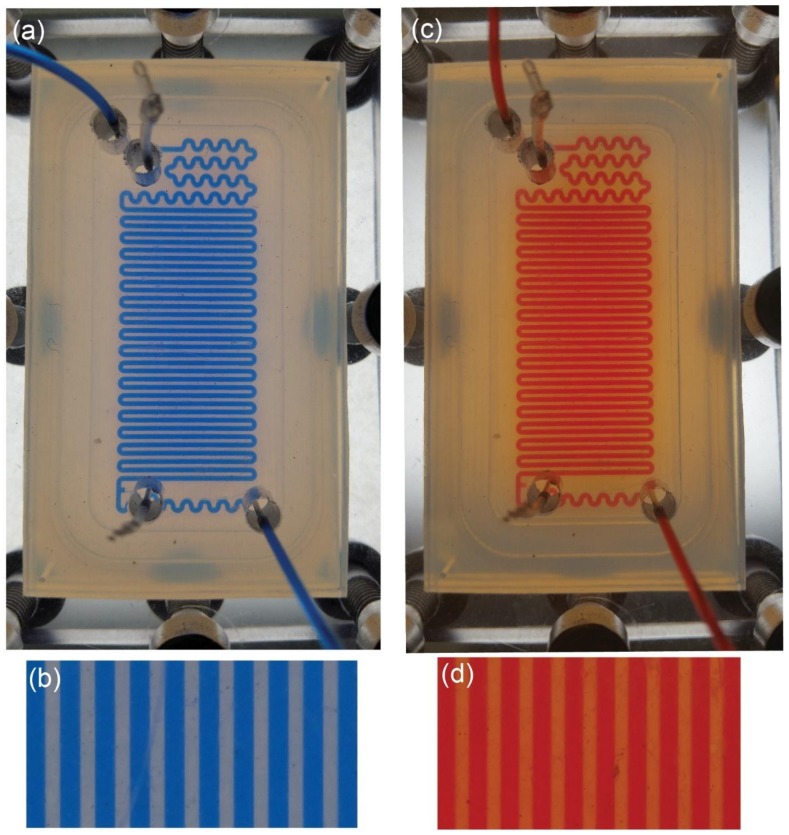
Visualization of the flow of EtOH with Oil Blue (**a**,**b**) and PrOH with Red Oil (**c**,**d**) in the photoreactor made of FEP. The photoreactor is placed between the holder made of 10 mm PC plates and compressed with M6 screws (visible at the edges of the pictures **a** and **c**). The flow of the organic solvents is 3 mL·h^−1^ which is typical for organic synthesis in microscale. There is no outflow of EtOH or PrOH outside the chip or to the mortise. The light color between main channels is a result of a thin layer of solvents between the two FEP plates (pictures **b** and **d**) due to capillary forces.

As was mentioned in Section 2.4, the microreactor must be sealed and no air or humidity can access the microchannel. We tested the tightness of flow inside the FEP microreactor with two organic solvents: ethanol (EtOH) with Oil Blue and isopropanol (PrOH) with Oil Red. The dyes are used for better contrast and improved visualization of the flow. We used a special holder made of polycarbonate (analogical in size and geometry of the opening to the holder made of non-transparent PE) to visualize the flow inside the chip. We connected the photoreactor to syringe pumps and filled microchannels with the typical velocity used in organic synthesis, e.g., 3 mL·h^−1^. Figure 4 depicts the microreactor filled with EtOH and PrOH, whereas Figure S8 demonstrates the experimental setup. 

Our tests demonstrated that there is no flow of EtOH or PrOH to the mortise, thus there is no outflow of the reagents or the product of reaction outside the microfluidic reactor. The pictures show a small change of color between the microchannels (bluish in case of EtOH and reddish in case of PrOH). We relate this change of color to the thin layer of liquid between the PEF plates due to capillary forces. Thickness of this film, in comparison to geometrical dimensions of the channel, is so small that it will not disturb the flow in the microchannel. Our measurements of the maximum working pressure demonstrated that our microreactor can be used up to 2.5 bar. 

### 3.4. Photochemical Synthesis 

**Figure 5:**
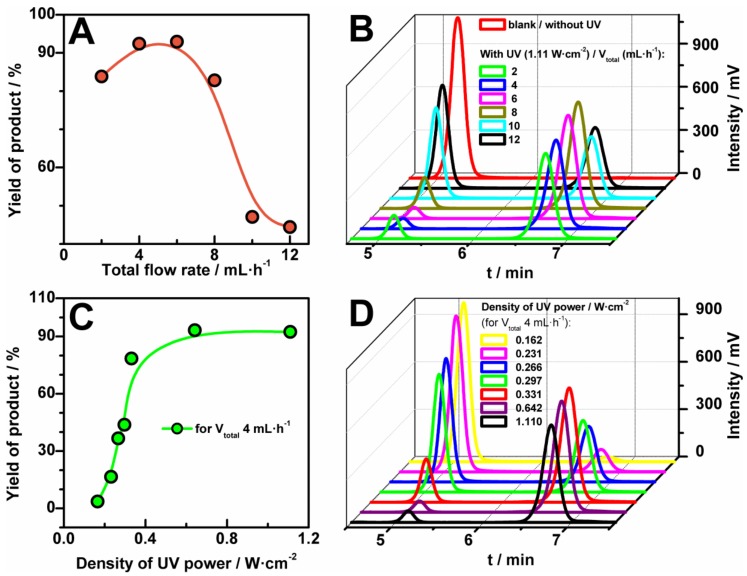
Yield of bromination reaction of 1-indanone (**1**, 0.25 M) in benzylic position with the use of N-bromosuccinimide (**2**, 0.26 M) in acetonitrile in the function of flow rate (**A**) and power of UV lamp (**C**) and the corresponding High Performance Liquid Chromatography (HPLC) chromatograms (**B**,**D**; eluent acetonitrile: water = 60:40). Reaction occurred at room temperature.

As a proof-of-concept of the use of the FEP microreactor we present two well-known [22,23] photochemical reactions (Figure 5). Both reactions should be carried out with the use of ultraviolet A (UVA) radiation (fluorescent black lights), which is typical and commonly used mostly for photochemical reactions. The first example of photochemical reaction is bromination of 1-indanone (**1**) in benzylic position with the use of N-bromosuccinimide (**2**) (NBS-Mediated Benzylic Bromination, Figure 1A) [22]. We tested this reaction in fixed conditions of temperature and solvent: 25 °C and acetonitrile, respectively. Moreover, the concentrations of reagents were also established and equal to 0.25 M and 0.26 M for 1-indanone and N-bromosuccinimide, respectively. Nevertheless, we examined various parameters for the photochemical bromination: different flow rates (Figure 5A,B) and power of UV source (Figure 5C,D). The progress of the reaction, i.e., its yield, was checked with the use of HPLC analysis and a UV detector. We observed two peaks in chromatograms (Figure 5B,D): first at 5.11 min characteristic for 1-indanone and second at 6.8 min correlated with the presence of the product, 3-bromoindan-1-one (**3**). The structure of the product was confirmed by nuclear magnetic resonance (NMR) analysis (Figure S9).

The tested range of total flow rates was from 2 mL·h^−1^ to 12 mL·h^−1^, where the flow was a sum of the flow rates of solutions: 1-indanone and N-bromosuccinimide in the fixed ratio 1:1. We found that the yield of bromination reaction depends strongly on the value of the flow rates (Figure 5A,B). As it can be seen, the yield increased with the increase of the flow up to 6 mL·h^−1^. It is worth noting, that for this value (6 mL·h^−1^) the conversion of substrates is maximum and is close to 90%. For a smaller value of the flow rate (2 mL·h^−1^) the yield of the reaction decreases slightly because of the generation of by-products. The decrease of the value of the flow rate is connected with the increase of UV exposed time of the sample in the microreactor and is connected with lower selectivity (see Figure S10).

In turn, a higher flow seems to reduce the time of the reaction, giving a significant decrease of the HLPC signal (Figure 5A). Furthermore, the bromination reaction was investigated for different values of power density of UV source—from 0 W·cm^−2^ to 1.11 W·cm^−2^. In Figure 5C, the yields of product versus the applied UV energy for the fixed flow rate, 4 mL·h^−1^, were plotted. The yield of the reaction increased very quickly with the increase of the UV power up to 0.5 W·cm^−2^, whereas after the value a steady-state stage was reached. 

**Figure 6:**
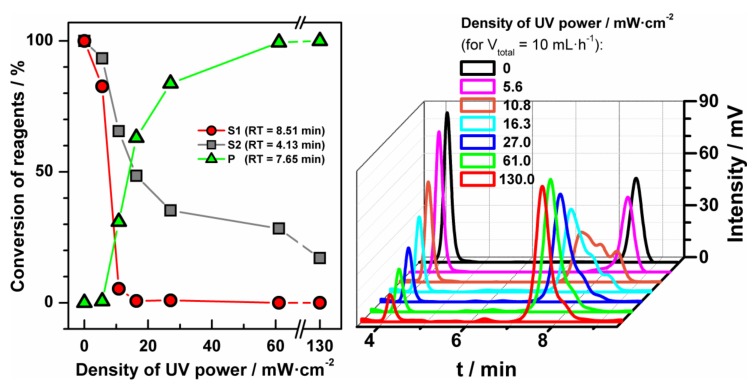
Conversion of reagents of the thiol-ene ‘click’ reaction in acetonitrile in the function of power of UV lamp and the corresponding HPLC chromatogram. Substrates were 2,4,6-triallyloxy-1,3,5-triazine (**4**) and, in excess, methyl mercaptoacetate (**5**). Reaction occurred at room temperature and yield receiving the product (**7**).

The second tested photochemical reaction was the thiol-ene ‘click’ reaction (Figure 5B): an addition reaction of methyl mercaptoacetate (0.80 M; **5**) to 2,4,6-triallyloxy-1,3,5-triazine (0.25 M; **4**) in acetonitrile [23]. Substrate **5** was used in triple excess because of the stoichiometry of the reaction (Figure 5B). Due to the improvement of selectivity and yield of the reaction we additionally used an organic photoinitiator, diphenyl(2,4,6-trimethylbenzoyl)phosphine oxide (**6**). It is worth underlining that the proper choice of photoinitiator enables us to use less energy for the initiation of the thiol-ene ‘click’ reaction. However, the employment of energy lower than 16 mW·cm^−2^ yields inadequate production of radicals and by-products can be formed (Figure 6). We tested this reaction for different power of UV source in fixed conditions of temperature and reagents concentrations with triple excess of thiol (Figure 6). The magnitude of conversion of the substrates depends on the power of UV source. Clearly, an increase of yield of the thiol-ene ‘click’ reaction is observed with the increase of the UV power, giving the full conversion at 60 mW·cm^−2^ and higher density of UV power, yielding product (**7**). The structure of product **7** was confirmed by NMR analysis (Figure S11).

The experimental setup, i.e., the FEP microreactor with mounted UV diode and controller and close-up of the microreactor between non-transparent plates is shown in Figure S7.

## 4. Conclusions

For organic photosynthesis involving UV irradiation, FEP is an ideal material due to its good transmission properties, excellent physical properties (e.g., the ability to withstand relatively high temperatures and mechanical stresses) and superb chemical resistance to organic solvents. Moreover, FEP is commercially available in a wide range of diameters; thus, its use in photo-reactors is cost-efficient. Therefore, FEP is an attractive material that can be used in the mass-production of microfluidic devices, that are dedicated to photochemical reactions. However, the level of transmittance (ca. 4% for wavelength 371 nm) may be too low for some photochemical reactions which need higher power density. This problem can be overcome with more powerful UV LED or a matrix of UV diodes, which should increase power density. Also, we can increase the length of the main channel, and thus increase the time of interaction of UV radiation with substrates.

Here, we have presented the use of an FEP microrector for photochemical reactions. Two different types of organic reaction: bromination and thiol-ene ‘click’ reactions were tested in the function of UV power and/or flow rate. We demonstrated that our FEP microreactor can be used in the laboratory during the photochemical reactions without the use of a fume hood or Schlenk line. Moreover, commonly-used FEP tubing reactors can be replaced by FEP compact microreactors that can be easily designed to the established photochemical reaction. The simplicity and versatility of our system makes our microreactor a potential new tool in every organic or metaloorganic laboratory. 

## Supplementary Materials

The following are available online at http://www.mdpi.com/2072-666X/9/4/156/s1, Figure S1: Experimental setup for measuring density of power; Figure S2: The density of power as a function of the power of the UV diode; Figure S3: The density of power as a function of the power of the UV diode, where the gap between UV diode and the sensor is simply an air, or fluorinated ethylene propylene (FEP) plates; Figure S4: CAD scheme of the FEP microreactor used in experiments; Figure S5: The experimental setup to measure the maximal working pressure; Figure S6: To test initial mixer we run acetonitrile and acetonitrile with a dye (Oil Blue); Figure S7: The experimental setup for photochemical reaction; Figure S8: Direct visualisation of the flow of EtOH with Oil Blue in FEP microreactor; Figure S9: The ^1^H nuclear magnetic resonance (NMR) spectrum of the product of bromination reaction; Figure S10: Complete chromatogram for bromination reaction for three different flow rates; Figure S11: The ^1^H NMR spectrum of the product of thiol-ene reaction.
