# Supplementary material for: An FEP Microfluidic Reactor for Photochemical Reactions
Source: Micromachines (Basel). 2018 Mar 30;9(4):156. doi: 10.3390/mi9040156 (PMC6187735; doi:10.3390/mi9040156)
Supplement: Supplementary file 1 [file micromachines-09-00156-s001.pdf]

# Supplementary Materials: An FEP Microfluidic Reactor for Photochemical Reactions

**Tomasz Szymborski, Paweł Jankowski, Dominika Ogończyk and Piotr Garstecki**

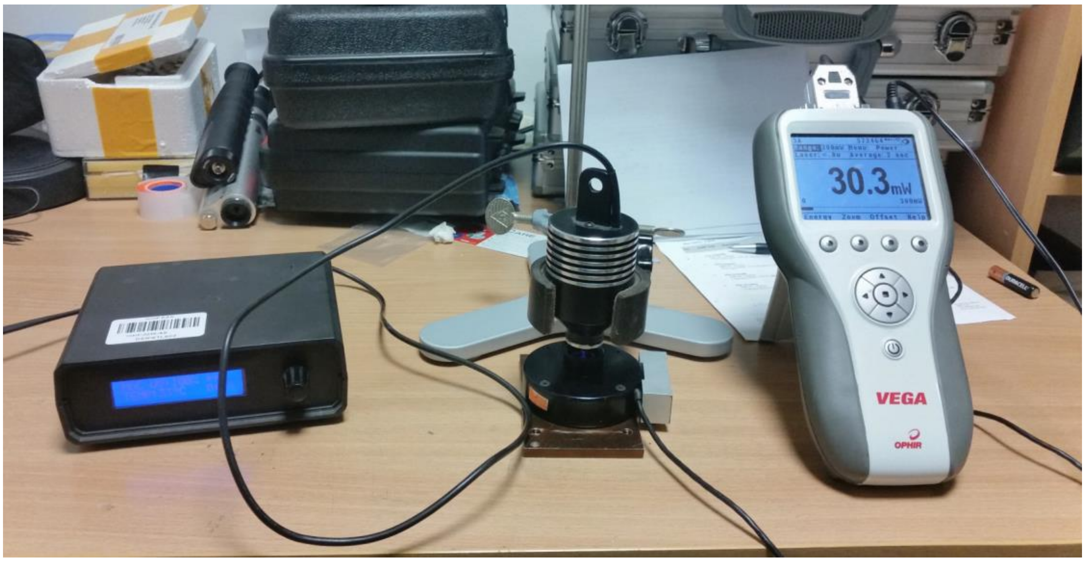


**Figure S1.** Experimental setup for measuring density of power. It consists of power meter (Orphir, Vega), detector and ultraviolet (UV) diode with home-made controller.

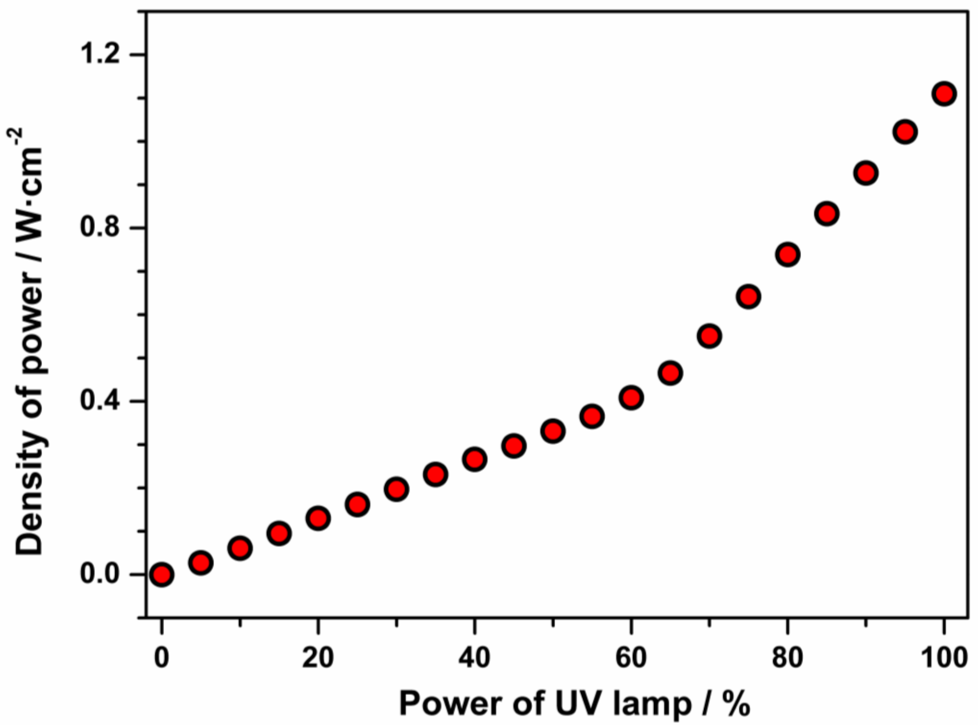


**Figure S2.** The density of power as a function of the power of the UV diode (scale in %). Measurements performed with power meter (Orphir, Vega, Ophir Optronics, Jerusalem, Israel) as shown in Figure S1.

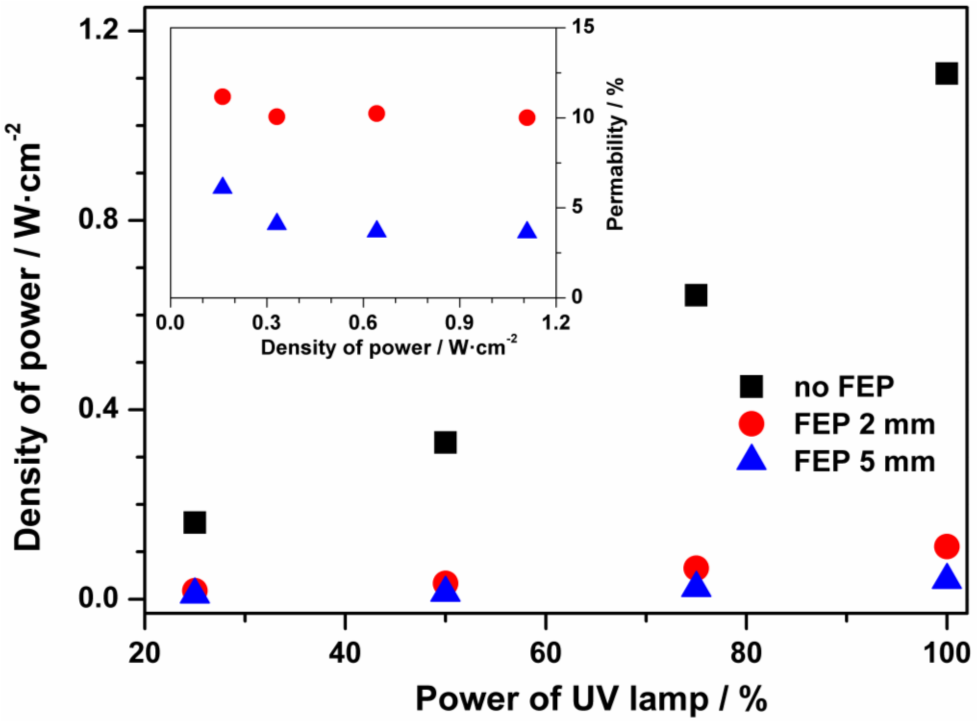


**Figure S3.** The density of power as a function of the power of the UV diode (scale in %), where the gap between UV diode and the sensor is simply an air, or fluorinated ethylene propylene (FEP) plates: 2 mm and 5 mm, respectively. Additionally, permeability in function of density of power for FEP plates is presented. This parameter is density of power normalized to the values adequate to power of UV lamp for "no FEP" (i.e. air gap) and was expressed as a percentage of these values.

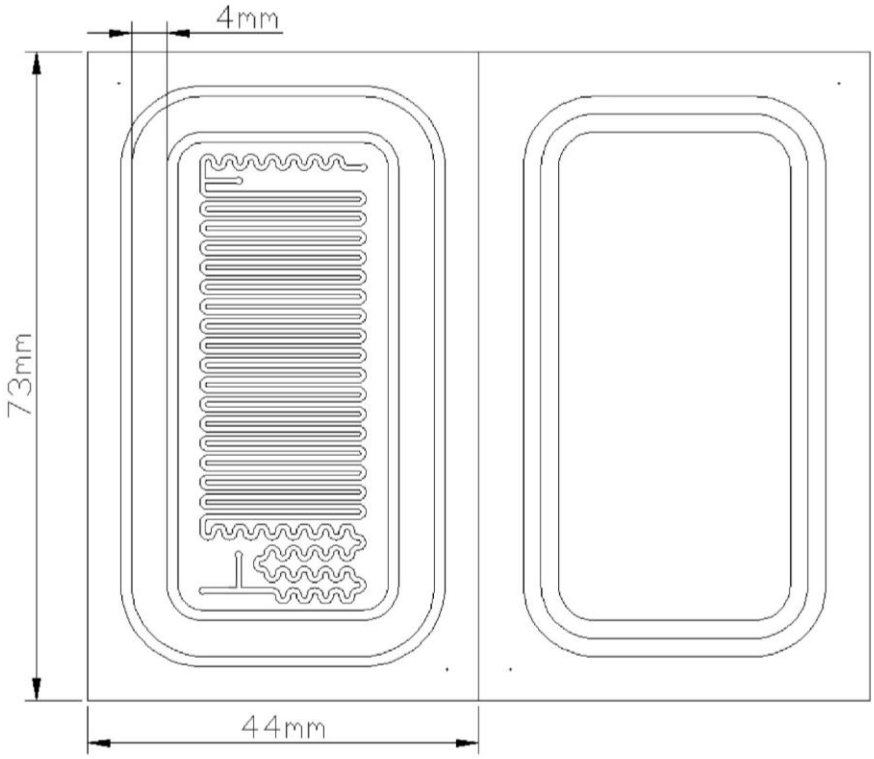


**Figure S4.** Computer aided design (CAD) scheme (from MasterCAM software, MasterCAM X2 , CNC Software, Inc., Tolland, CT, USA) of the FEP microreactor used in experiments. Left part demonstrate male part with microchannels and tenon, whereas right part demonstrates a female plate with a groove (left and right line is the edge of the groove whereas middle line is a trajectory of the milling tool).

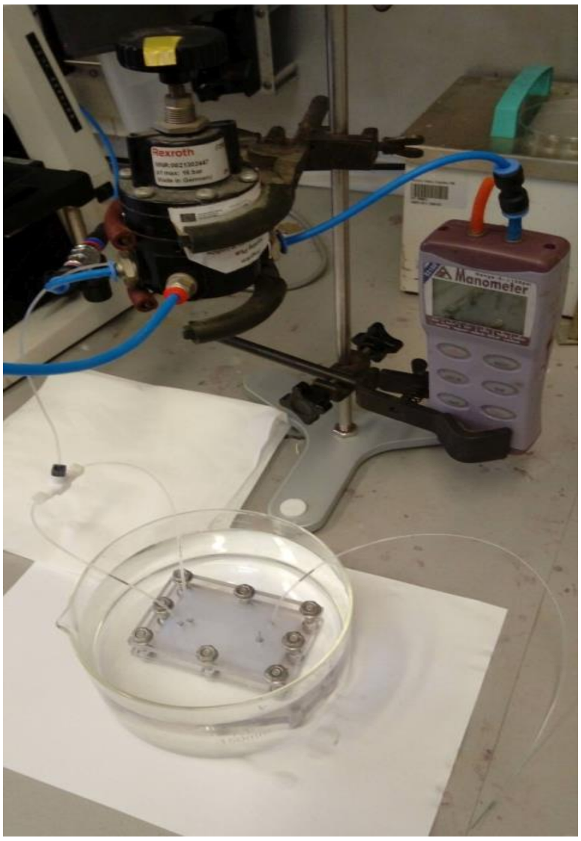


**Figure S5.** The experimental setup to measure the maximal working pressure. We placed the microreactor into water filled beaker and closed the outlet whereas we pushed compressed air through the inlets. We monitored pressure via manometer and increased the pressure till we observed the unsealing of the chip, i.e. bubbles of the air. The maximal working presssure is 2.5 bar.

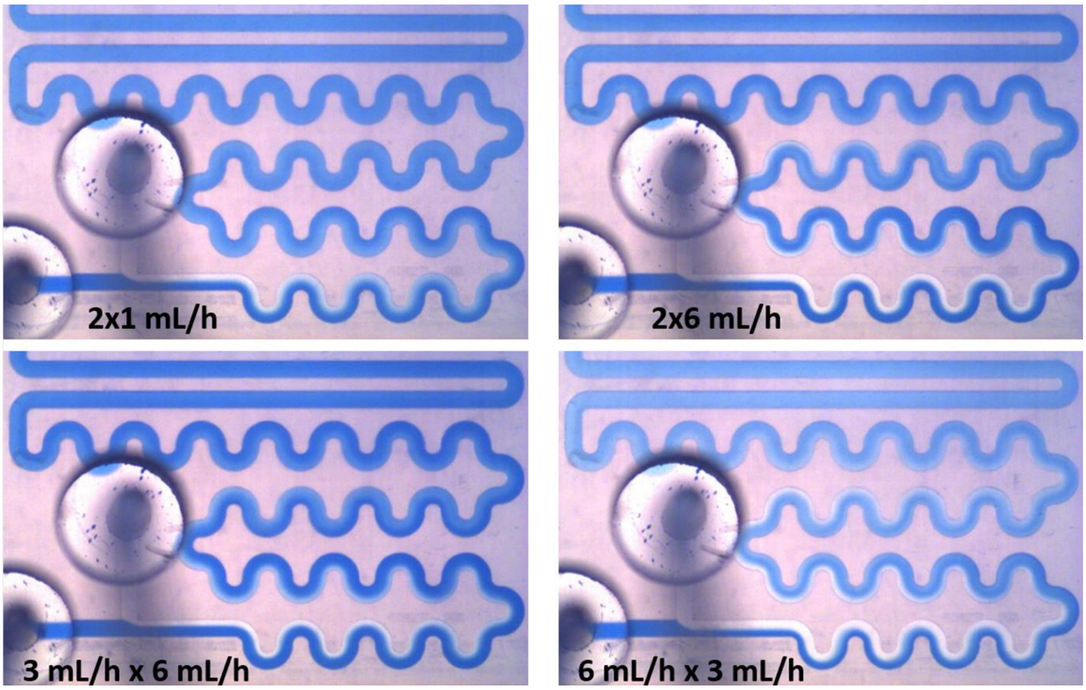


**Figure S6.** To test initial mixer we run acetonitrile and acetonitrile with a dye (Oil Blue). Top row show equal flow: 1 ml/h in each channel on left and 6 ml/h in figure on right. Pictures in bottom row demonstrate mixing for asymmetrical flow. In all cases the two streams mix perfectly before they reach main channel.

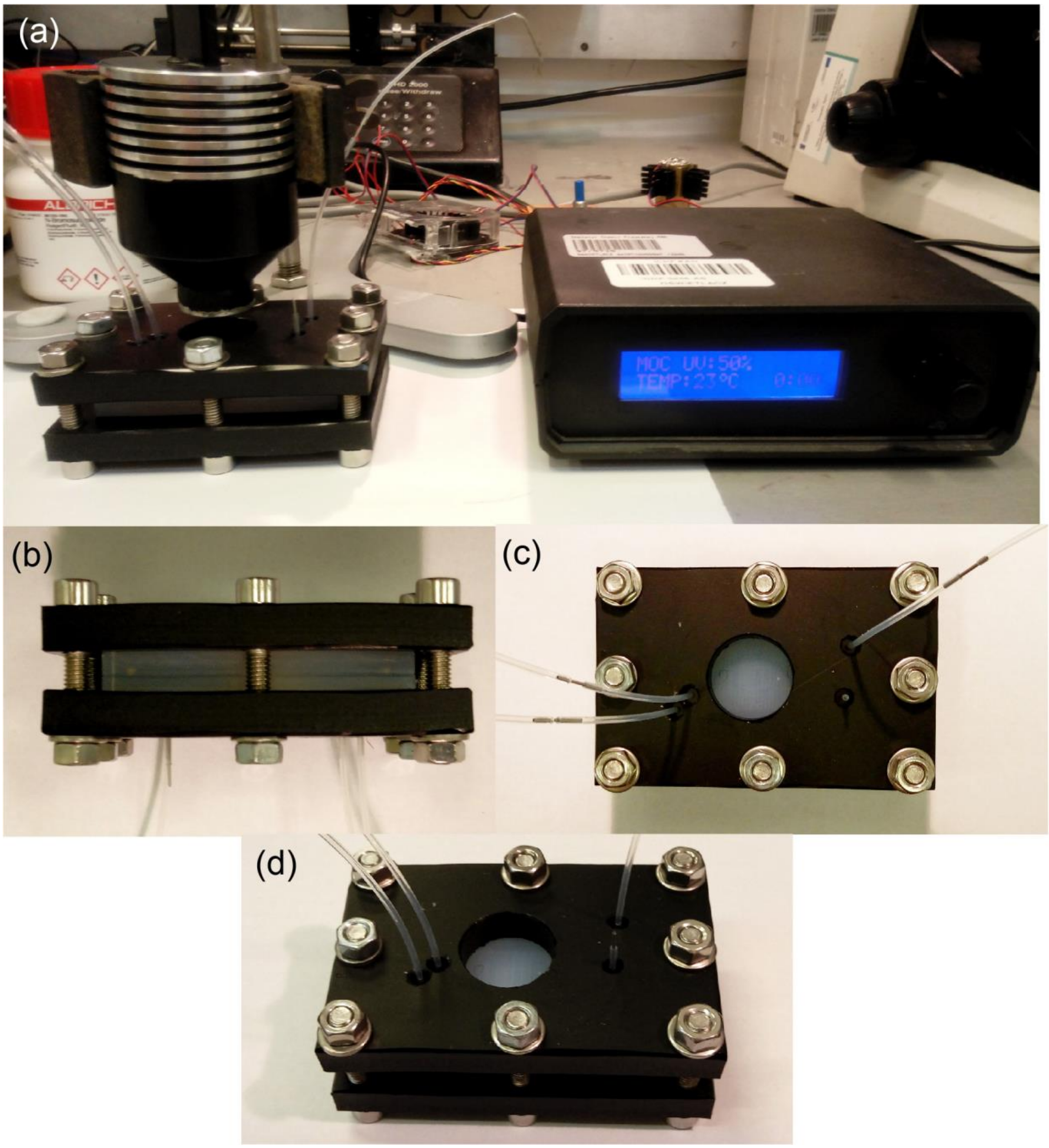


**Figure S7.** The experimental setup (**a**) for photochemical reaction consists of FEP microreactor placed between two non-transparent 10 mm thick plates, compressed with eighth M6 screws. The UV diode is placed in circular opening micromachinned in top plate. The power of UV diode is regulated via home-made controller. The top plate consists of four small holes for FEP tubings (**c** and **d**).

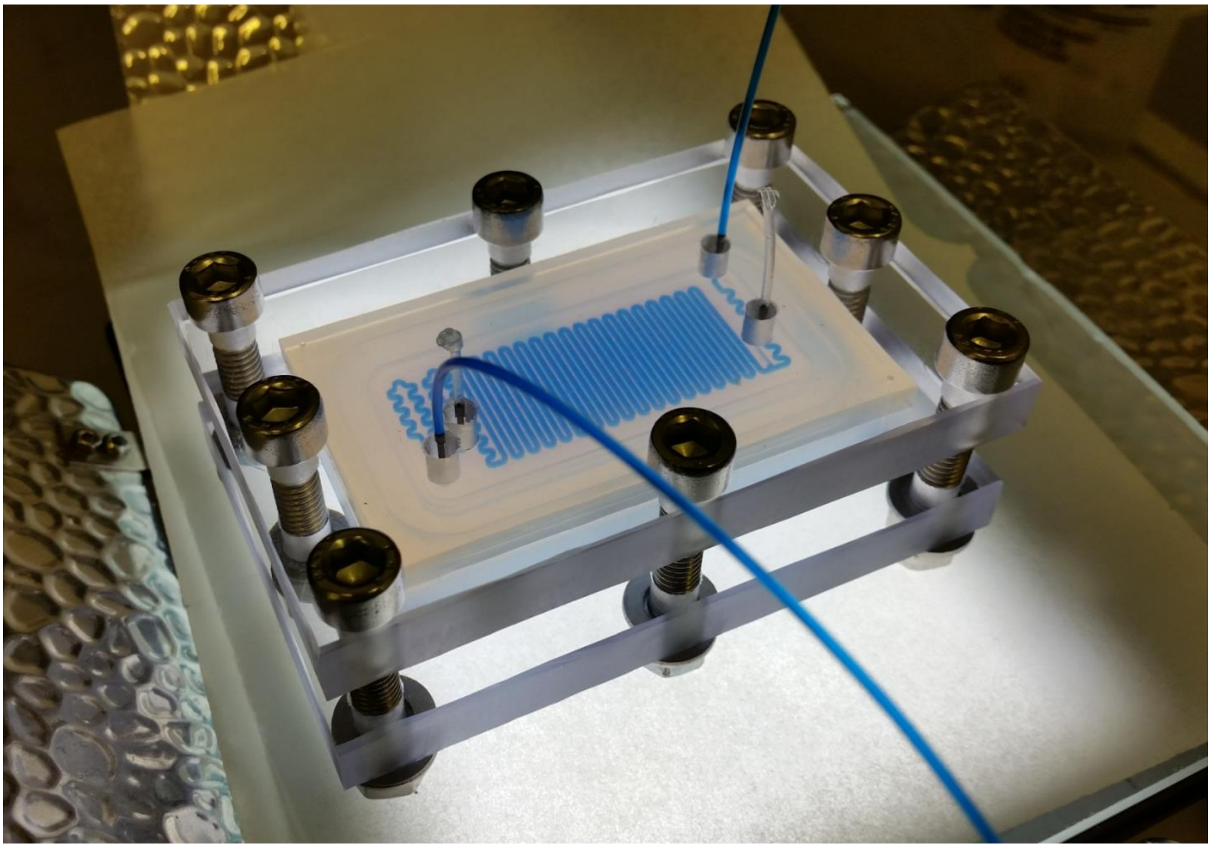

**Figure S8.** Direct visualisation of the flow of EtOH with Oil Blue in FEP microreactor. The microreactor is placed between two polycarbonate (PC) plates and compressed with eighth M6 screws. The flow of the ethanol with oil Blue is maintained with syringe pump.

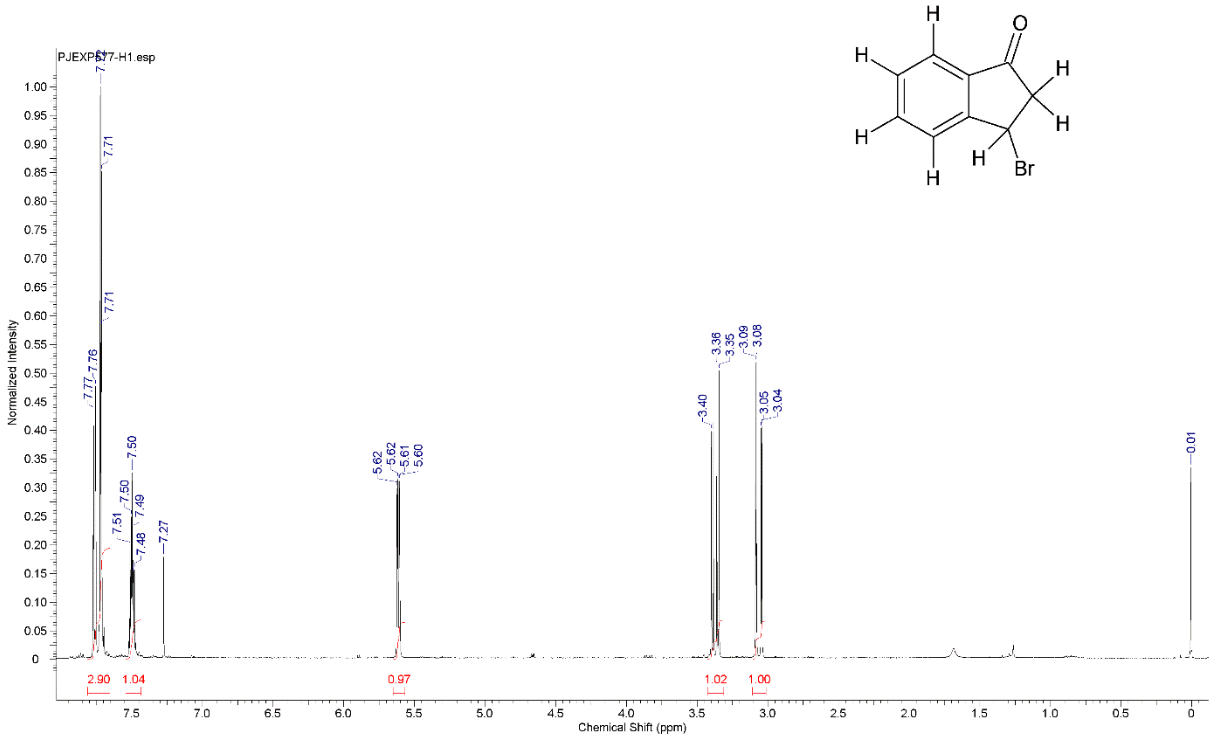


**Figure S9.** The $^{1}$H nuclear magnetic resonance (NMR) spectrum of the product of bromination reaction (crude reaction product after filtration through silica gel).

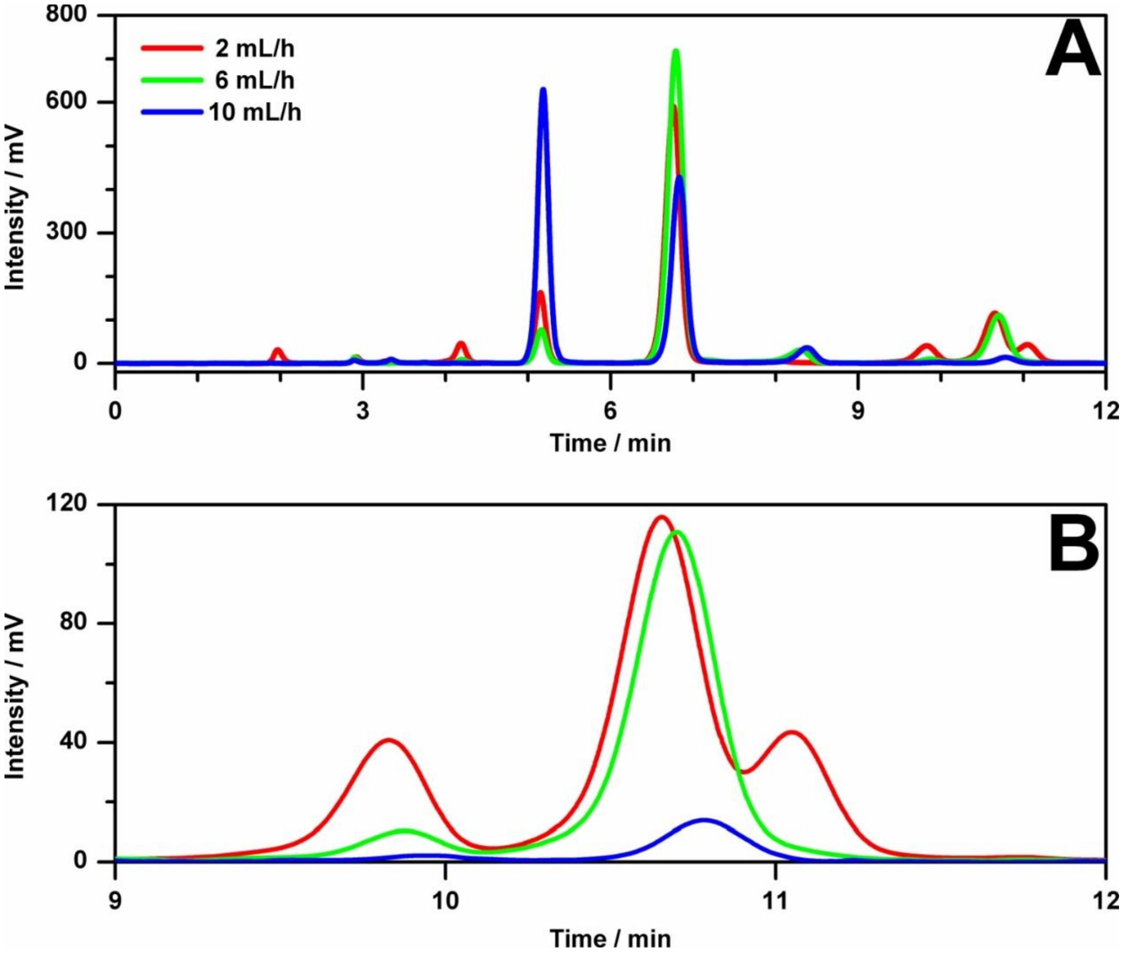


**Figure S10.** Complete chromatogram for bromination reaction for three different flow rates (**A**). In the inset (**B**), the part of chromatogram for different by-products with various amounts are presented. Decrease of value of flow rate is connected with increase of UV exposed time of sample in microreactor and is connected with lower selectivity (the yield is lower).

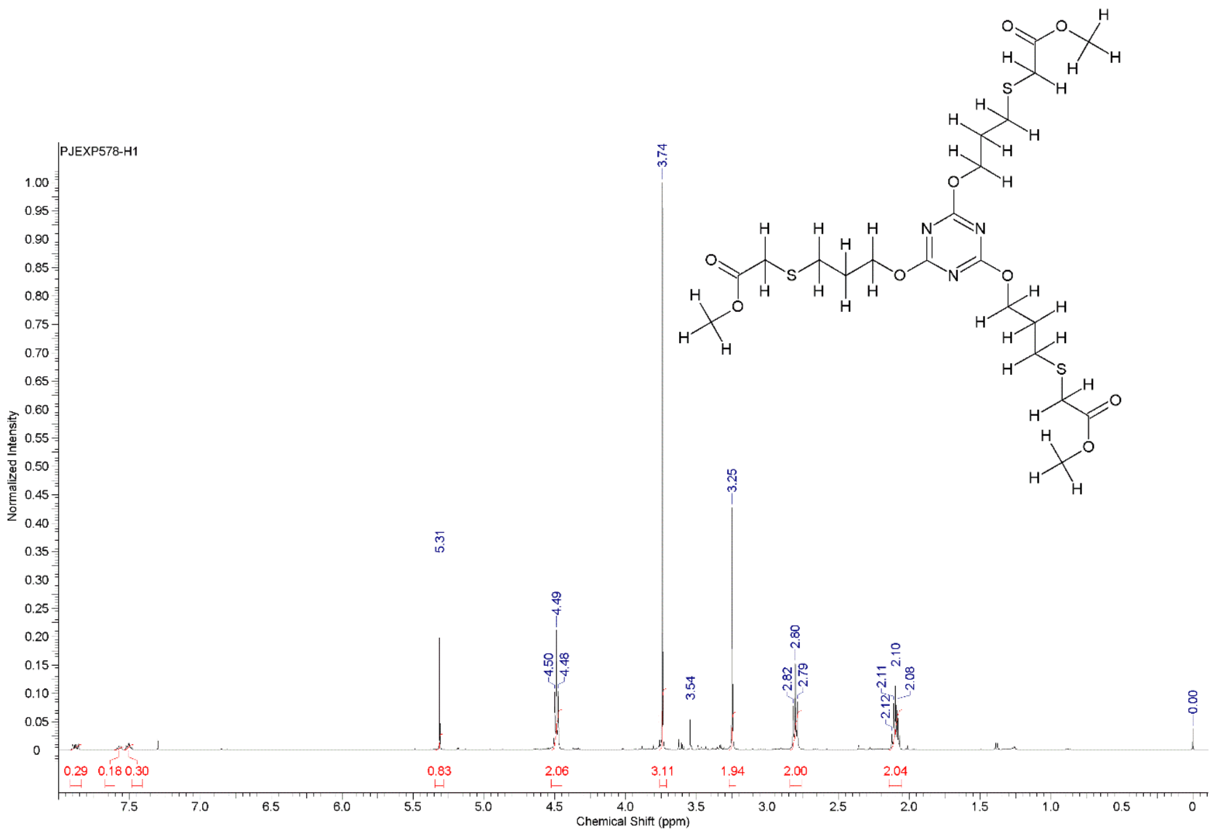


**Figure S11.** The $^{1}$H NMR spectrum of the product of thiol-ene reaction (crude reaction product after filtration through silica gel).

**3-bromoindan-1-one (3).** $^{1}$H NMR (500 MHz, $CDCl_3$): δ = 7.76 (d, J = 7.7 Hz, 1H), 7.71-7.72 (m, 2H), 7.48-7.51 (m, 1H), 5.61 (dd, J = 2.5, 7.2 Hz, 1H), 3.38 (dd, J = 7.2, 19.8 Hz, 1H), 3.07 (dd, J = 2.7, 19.8 Hz, 1H). $^{13}$C NMR (500 MHz, $CDCl_3$): δ = 204.13, 156.92, 138.64, 138.26, 132.32, 130.17, 126.04, 50.74, 43.30.

**Product of thiol-ene reaction (7).** $^{1}$H NMR (500 MHz, $CDCl_3$): δ = 4.49 (t, J = 6.2 Hz, 6H), 3.74 (s, 9H), 3.25 (s, 6H), 2.80 (t, J = 7.2 Hz, 6H), 2.07-2.12 (m, 6H). $^{13}$C NMR (500 MHz, $CDCl_3$): δ = 172.87, 170.65, 66.53, 52.32, 33.30, 28.93, 27.87.
